# The lysosomal trafficking regulator “LYST”: an 80-year traffic jam

**DOI:** 10.3389/fimmu.2024.1404846

**Published:** 2024-05-07

**Authors:** Mackenzie E. Turner, Jingru Che, Gabriel J. M. Mirhaidari, Catherine C. Kennedy, Kevin M. Blum, Sahana Rajesh, Jacob C. Zbinden, Christopher K. Breuer, Cameron A. Best, Jenny C. Barker

**Affiliations:** ^1^ Center for Regenerative Medicine, Abigail Wexner Research Institute at Nationwide Children’s Hospital, Columbus, OH, United States; ^2^ Molecular and Cellular Developmental Biology Graduate Program, The Ohio State University, Columbus, OH, United States; ^3^ The Ohio State University College of Medicine, Columbus, OH, United States; ^4^ Department of Plastic and Reconstructive Surgery, The Ohio State University Medical Center, Columbus, OH, United States

**Keywords:** lysosomes, vesicle traffic, Chédiak-Higashi syndrome, cancer, wound healing, immunotherapy, LYST, beige

## Abstract

Lysosomes and lysosome related organelles (LROs) are dynamic organelles at the intersection of various pathways involved in maintaining cellular hemostasis and regulating cellular functions. Vesicle trafficking of lysosomes and LROs are critical to maintain their functions. The lysosomal trafficking regulator (LYST) is an elusive protein important for the regulation of membrane dynamics and intracellular trafficking of lysosomes and LROs. Mutations to the LYST gene result in Chédiak-Higashi syndrome, an autosomal recessive immunodeficiency characterized by defective granule exocytosis, cytotoxicity, etc. Despite eight decades passing since its initial discovery, a comprehensive understanding of LYST’s function in cellular biology remains unresolved. Accumulating evidence suggests that dysregulation of LYST function also manifests in other disease states. Here, we review the available literature to consolidate available scientific endeavors in relation to LYST and discuss its relevance for immunomodulatory therapies, regenerative medicine and cancer applications.

## 1 The lysosomal trafficking regulator: the roadmap

In 1943, a pediatrician discovered a strange, previously uncharacterized disease. The pediatrician described the patients’ peripheral blood smears as containing abnormally large, distended granules, a first-in-man observation (1, 2). Clinicians in Cuba and Japan corroborated these findings, reporting similar childhood cases unified by three hallmark features: oculocutaneous albinism, granule abnormality, and death in early childhood (3, 4). This disease came to be known as Chédiak-Higashi Syndrome (CHS) (5, 6). In the ensuing decades, the lysosomal trafficking regulator (*LYST*) has emerged as the gene underlying CHS. While LYST initially garnered attention due to its connection to CHS, researchers identified LYST as a pivotal player in cellular and immune functions. Discovering CHS marked the beginning of decades of efforts to understand LYST’s role in biology and eventually its implications for human health and disease.

For decades following the landmark discovery, the scientific community was puzzled by this abnormal disease. While no one understood the etiology of disease, it was clear that CHS caused immune dysfunction and granule anomalies that warranted further investigation. These investigations eventually led to identifying the enlarged intracellular inclusions as being lysosomal in origin (7–9). Following the discovery of CHS, similar phenotypes were reported in animal models (**Table 1**). Many seminal publications that provide insights into LYST molecular function involve the mouse homolog of LYST, beige, which has a phenotype that closely resembles human CHS. This model is discussed in more detail in section III. Animal models of LYST mutations. Human samples and animal models provided tools to examine the consequences of CHS on immune function and molecular biology, but efforts were limited by the unidentified cause of CHS. It was not until 1996, forty years after the first description of CHS, that researchers identified and cloned the causative human *LYST* gene (19, 20), which consists of 53 exons and is located on chromosome 1q42.1-1q42.2 (21).

**Table 1:** Summary of the model organisms available for lyst research.

Animal	Genetic Variant	Mutation Details	Phenotypic Observations	Ref
***Mouse***	Beige (*Lyst* ^bg^); Beige-J (*Lyst* ^bg-J^); Beige-2J (*Lyst^bg-2J^*)	Lack WD40 and BEACH	- Light coat color - Delayed wound healing - Defective natural killer cell cytotoxicity - Reduced chemotaxis and bactericidal activity of granulocytes - Decreased T-cell response to allogeneic tumor cells	(10)
	Beige-grey (*Lyst* ^bg-grey^)	Unstable protein	- Light coat color	(11)
	*Lyst* ^Ing3618^	Missense WD40 mutation	- Enlarged lysosomes in CNS and PNS - Purkinje cell degeneration + severe neurological degeneration - Prolonged bleeding; mild hypopigmentation	(12)
***Drosophila melanogaster***	*Mauve* mutation	Nonsense mutation in mv2 (3L-85) allele	- Enlarged pigment granules with decreased number - Defective cellular immunity + increased susceptibility to infection - Enlarged starvation-induced autophagosomes - Eye color defect	(13)
***Caenorhabditis elegans***	*Lyst-1* mutant	*lyst*-1(gk295717); *lyst*-1(gk803491)	- Decreased size and increased number of gut granules - Reduced lysosome size - Decreased number of early endosomes	(14)
***Dicyostelium discoideum***	*LvsB* null mutant	*LvsB (-/-)* mutant	- Secretory defect - Defective lysosome maturation; reduced number of post lysosomes - Normal development	(15)
***Corn snake***	*Lavender* variant	Exon 41 SNP ➔ truncated BEACH & partially absent WD40	- Enlarged and aggregated LROs - Reduced chromatophores, gray and pink color morph	(16)
***Japanese black cattle***	Bovine *Lyst* missense	A ➔G at nucleotide 6050 ➔ H2015R	- Hypopigmentation - Heritable bleeding disorder	(17)
***Zebrafish***	*Lyst 7653 ^mu107^*	C ➔T mutation at nucleotide 987	- Hypopigmentation - Hepatomegaly + steatosis - Kidney defects	(18)

After identifying the causative gene, researchers began dedicating efforts to understanding a place for LYST in cellular biology. Some groups focused on clarifying whether LYST causes granule abnormalities through fusion or fission processes (8, 22–28). Other groups were interested in potential binding partners of LYST that could suggest how LYST is involved in membrane dynamics and vesicle trafficking (29, 30). The journey to understanding LYST has extended into the 21^st^ century. With time, more tools have become available for studying LYST function. Recently, sequencing and structural analysis provided a more detailed molecular understanding of the LYST gene and protein structure (31, 32). These advancements provided more clues for its involvement in a myriad of cellular processes. Over the past decade alone, LYST has been linked to human health outside of CHS through connections to cancer and wound healing (33–40).

Despite longstanding interest in investigating LYST, a complete understanding of LYST biology is yet to be established. Here, we provide a comprehensive literature review to consolidate available scientific endeavors in relation to LYST, which may be of great value to the basic science and clinical community.

## 2 LYST and Chédiak-Higashi syndrome

Following the initial discovery of the CHS, researchers published additional reports discussing clinical cases. The connection of *LYST* to CHS prompted further efforts to understand LYST function, which ultimately revealed its involvement in cellular biology and human health outside of CHS-related conditions. This autosomal recessive disorder presents along a clinical spectrum characterized by hypopigmentation, recurrent bacterial infections, developmental delays, and coagulation defects (41–43). Over 80% of patients reach the “accelerated phase” of the disease, wherein they develop life-threatening hemophagocytic lymphohistiocytosis (HLH), a hyper-inflammatory disorder associated with recurrent viral infections. This phenomenon represents the most common cause of death in patients with CHS (1, 2). Hematopoietic stem cell transplant is the only available curative treatment for CHS. Case studies demonstrated its potential in correcting immunologic complications and extending lifespan into adulthood (44). Yet, patients who survive into adulthood develop progressive central and peripheral neurodegeneration. A paucity of literature exists surrounding CHS, and while the exact prevalence is unknown, there have been fewer than 500 reported cases worldwide since its discovery (45, 46).

## 3 Animal models of LYST mutations

Animal models are critical for gaining further insight into the pathophysiology of LYST mutations. The most investigated animal models are murine equivalents of CHS. A radiation-induced mutation resulted in the first described *Lyst*-mutant mouse, deemed the beige (bg or *Lyst* ^bg^) mouse for its silvery-grey coat coloration compared to C57BL/6J wild-type (WT) mice (47, 48). The spontaneous mutation of beige allele is caused by either i. 3-bp deletion of a single amino acid (isoleucine) within WD40 domain, or ii. a LINE1 element insertion, resulting in a truncated Lyst protein lacking both WD40 and BEACH domains (49). The genetic details are more thoroughly described in section VII toward a molecular understanding of LYST. Today, researchers use the beige-J (*Lyst* ^bg-J^) strain, which is a spontaneous re-mutation virtually identical to the originally reported *Lyst* ^bg^ mice (10). *Lyst* ^bg-J^ and the less commonly used *Lyst* ^bg-2J^ mice, are phenotypically identical to the original *Lyst* ^bg^ mouse (10). Murine beige-grey mutant (*Lyst* ^bg-grey^) mice carry a chemically induced *Lyst* mutation (discovered in an ENU mutation screen) that results in an unstable Lyst protein caused by Exon 25 skipping (11). Thus, unlike *Lyst* ^bg^ and *Lyst* ^bg-J^ mice, the beige-grey mutation leaves the WD40 and BEACH domains intact (11). Notably, all *Lyst-*mutant mice exhibit phenotypes commonly seen in human CHS patients, including abnormal pigmentation of coat color comparable to oculocutaneous albinism in human, immunodeficiency associated with abnormal or defective NK cells, and enlarged granules (**Table 1**). The differential inclusion and/or exclusion of WD40 repeats, the BEACH domain, and Exon 25 result in a variety of *Lyst*-mutant transcripts and a spectrum of unstable Lyst proteins, potentially mirroring the spectrum of disease severity observed in human CHS patients.

A murine missense mutation (*Lyst* ^Ing3618^) located in Exon 48 of the WD40 domain caused a progressive neurodegenerative phenotype, and the mouse lacked the immunodeficiency observed in beige mice (12). *Lyst* ^Ing3618^/*Lyst* ^Ing3618^ mice display progressive Purkinje cell degeneration and impaired neurological functions during aging compared to WT mice (12). By 18 months, the *Lyst* ^Ing3618^ mice have enlarged lysosomes and almost no detectable neuronal Purkinje cells. This phenotype resembles the mild, atypical forms of CHS, characterized by early neurodevelopmental issues in childhood followed by progressive neurodegeneration in adulthood (50). Many other animal models possessing CHS-equivalent diseases resulting from *Lyst* mutations have also been reported (**Table 1**), including mink (51, 52), cat (53–55), fox (56, 57), Wagyu cattle (58), corn snake (16), and rat (59).

Together, the beige mutant mice and related strains present a compelling experimental paradigm wherein the relative contributions of intact WD40 or WD40/BEACH domains to Lyst-dependent mechanisms could be interrogated *in vivo* to better understand LYST function in humans. We anticipate that these models could not only be used for understanding CHS pathology but also for investigating defective vesicular trafficking.

## 4 LYST activity in specific cell types

**Figure 1:**
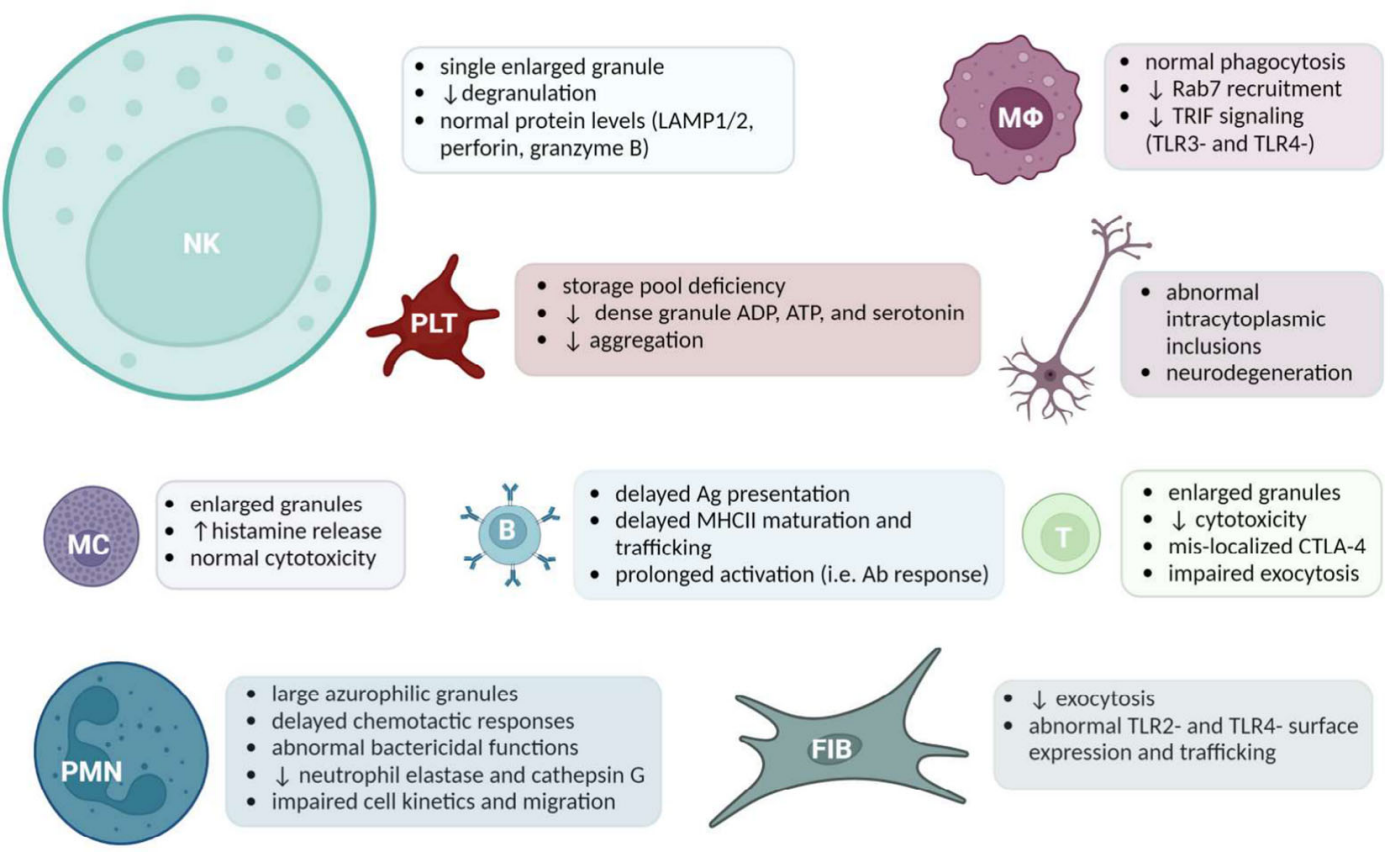
A Summary of Cell Specific LYST-Deficient Phenotypes. Acronyms (from left to right): Natural killer (NK) cells, Macrophages (MΦ), Platelets (PLT), cells of the nervous system, Mast cells (MC), B cells, Cytotoxic T cells, Polymorphonuclear leukocytes (PMN), Fibroblasts (FIB). The size of the cell reflects the amount of literature available. Created with BioRender.com.

Much of what is known about LYST comes from the cell type-specific manifestations of LYST dysfunction observed in disease. LYST underlies the vesicle trafficking responsible for mobilizing specialized LROs that are central to host defense. In parallel with characterization of Lyst-dependent molecular mechanisms, investigations into the cell type-specific mechanisms demonstrated the extensive manifestations of defective endolysosomal trafficking. Enlarged granules have been observed in *LYST*-mutant NK cells (60), cytotoxic T cells (61), B cells and helper T cells, neurons, neutrophils/granulocytes, monocytes/macrophages, fibroblasts, platelets, and pigment-producing cells. Available evidence regarding each cell type is discussed in the following section, a graphical overview of which is provided in **Figure 1**.

### 4.1 Natural killer cells

Defective natural killer (NK) cell function is a common feature of CHS. NK cells are a lymphocyte subset critical to the innate host defense against tumors and microbial infections (62). The cytotoxic function of NK cells is accomplished via secretory lysosome exocytosis. In this process, cytotoxic proteins are released from the NK cell secretory lysosome, or lytic granule, resulting in the death of the target cell through interruption of membrane integrity and inducing apoptosis (62, 63). Interestingly, NK cells are present in normal quantities in CHS patients and the *LYST* mutation did not impact their ability to recognize and bind target cells *in vitro* (61). Studies involving CHS patient-derived cells suggested that LYST may modulate the exocytosis of secretory lysosomes (64). However, LYST-deficient granules contained a relatively normal number of lysosome-associated proteins and lytic protein activity, despite their enlarged morphology (60). Chiang et al. reported that enlarged granules failed to polarize to the immune synapse and release their cytotoxic cargo (61). To further resolve the impaired transportation of enlarged granules, Gil-Krzewska et al. investigated the actin meshwork as a potential mechanistic barrier to polarization (60). They observed that the cellular actin meshwork is not permissive for enlarged granules in LYST-deficient cells, since lytic granules must navigate the tangled actin network at the immunologic synapse to be secreted onto target cells (60). The authors concluded that the cortical actin meshwork may prohibit the exocytosis of *LYST*-mutated granules in NK cells. Unresponsive NK cells increase the patient’s susceptibility to contracting viral infections (**Figure 1**) which are known to accelerate CHS to HLH.

### 4.2 Cytotoxic T cells

Cytotoxic T lymphocytes (CTLs) have an important role in the adaptive immune response, recognizing antigens presented in MHC class I molecules (65). Similar to NK cells, activated CTLs kill target cells through the release of perforin and granzyme from lytic granules (66). LYST deficiency reduces the normal cytotoxicity of CTLs and enlarges their granules (61, 67, 68).

Compared to CHS NK cells, *Lyst*-mutant CTLs contain a greater quantity of relatively smaller yet still abnormally enlarged LROs. This suggests that these smaller CTL granules may more efficiently navigate cellular obstacles. However, *in vitro* assays revealed a marked deficiency of granule exocytosis and decreased cytotoxicity (61). As described previously, while entanglement in the cytoskeleton may impact the exocytosis of enlarged LROs, data from CHS CTLs suggest that the mechanism of defective vesicular transport is likely more complex (69).

Cytotoxic T lymphocyte-associated antigen 4 (CTLA-4), a major T cell regulator, functions by inhibiting the activation of T cells. Although CTLA-4 is mainly stored in intracellular vesicles, cell membrane expression is highly regulated by endocytosis and trafficking through a secretory lysosome pathway. An earlier study revealed that CHS patients have impaired intracellular trafficking of CTLA-4 in the T cells, which results in defective cell-surface expression of CTLA-4 (69). This abnormal regulation of cytotoxic T cells likely contributes to the accelerated phase of CHS (69).

### 4.3 B cells and T helper cells

Although efforts to define the nature and extent of the LYST protein’s role in B and T helper cell (Th cells) biology have provided some insights regarding the kinetics of adaptive immune system activation in CHS, recent mechanistic investigations are lacking. CHS CD4^+^ T cells had shown decreased infection compared to WT CD4^+^ T cells upon HIV-1 exposure, which primarily spreads through cell-to-cell contact involving virion fusion with cell membrane (70). Further analysis indicated that HIV-1 spread is restricted by deficient LYST function in CHS T cells.

B cells are dependent on endosomal trafficking pathways to internalize and process antigens for major histocompatibility complex class II (MHCII) mediated surface presentation (71). *LYST* mutations slow the kinetics of antigen presentation and MHCII maturation and transport (72, 73). In beige B cells, one study observed delayed mobilization of the endocytosed antigen-B cell receptor (Ag-BCR) to lysosomes, which led to the suboptimal presentation of BCR-targeted antigen to T cells *in vitro* (73). The authors describe how a delay in antigen presentation results in prolonged B cell activation, even in the presence of low antigen levels (73). Sustained B cell engagement may reinforce pro-proliferative signaling cascades, and when uncontrolled, lead to a constant state of lymphoproliferation as demonstrated in the “accelerated phase” resulting in HLH (74, 75). Indeed, HLH is caused by defective vesicle trafficking-dependent immune cell apoptosis, leading to marked overactivation of host defenses, bone marrow infiltration, pancytopenia, liver dysfunction, and hepatosplenomegaly often requiring cytotoxic immunosuppression or stem cell transplant.

### 4.4 Cells of the nervous system

While CHS is primarily described by severe immunological defects, neurologic impairments are also commonly observed among mild to late-onset CHS patients. Patients affected by this condition may experience developmental impairment (learning disabilities), dementia, progressive neuronal degeneration (especially among those surviving to adulthood), seizures, sensory deficit, and weakness (50).

A case study published in 1994 reported a 39-year-old CHS patient who developed Parkinsonian features along with dementia. Single-photon emission computerized tomography (SPECT) revealed severe neuronal degeneration in the cortex, basal ganglia, brainstem, and spinal cord (9). Sung et al. reported abnormal intracytoplasmic inclusions in various CHS patient cells including neurons, astrocytes, epithelial cells of the choroid plexus, satellite cells of the dorsal spinal ganglia, and Schwann cells (76). A comparable phenotype has also been described in mice. As mentioned in the III. Animal models of LYST mutations section, the *Lyst* ^Ing3618^ mouse (**Table 1**) exhibits a neurodegenerative phenotype without immunological defects (12). This represents the only immune-competent Lyst-mutant murine model reported to date, offering a window into the relatively unexplored biology of CHS patients with neurological impairments. The effects of the *LYST* mutation on the central nervous system requires further exploration.

### 4.5 Neutrophils

Neutrophils are a subset of granulocytes critical to the detection and elimination of microbes and foreign bodies. Neutrophils circulate in a quiescent state until activated by cytokines and growth factors released from sentinel cells such as endothelial cells (77, 78). Once activated, neutrophils degranulate, or release stored pro-inflammatory mediators and/or microbicidal proteins, from either: i. primary or azurophilic, ii. secondary or specific, iii. tertiary, or iv. secretory vesicle granular subset (79). Neutrophils isolated from CHS patients demonstrated delayed chemotactic responses, large and irregular azurophilic granules, and abnormal bactericidal functions (80). Azurophilic granules contain potent hydrolytic enzymes that allow the neutrophil to confer bactericidal and cytotoxic properties (81). CHS patients were deficient in neutrophil elastase and cathepsin G (82). The deficiency of azurophilic components may be partially responsible for the microbicidal defect observed in LYST-deficient cells. The presence of the enlarged, abnormal granules in CHS neutrophils also impaired cell kinetics and prohibited proper migration (25, 83). While the observed neutrophil defect does not appear to impact signal recognition, *LYST* mutations among CHS patients likely interfere with vesicular trafficking pathways. Such pathways direct granulocyte migration including cytokine release by macrophages or signaling by endothelial cells (83–85). Neutrophils represent a prototypical LYST-dependent cell type, wherein interrupted antimicrobial function due to abnormal phagolysosomes renders patients and laboratory animals alike susceptible to bacterial infections. The disruption of normal antimicrobial function likely results in recurrent infections of CHS patients. The correlation between abnormal neutrophil function and clinical manifestations raises an intriguing possibility that recent advances in neutrophil biology and therapeutics could yield targeted immunomodulatory therapies.

### 4.6 Fibroblasts

Fibroblasts have an essential role in wound healing, tissue regrowth, and regulating immune responses through secretion and remodeling of extracellular matrix (ECM) (86).. CHS and beige fibroblasts contain a relatively reduced quantity of abnormally enlarged lysosomes (22, 87). With their wide availability, ease of culture, and well-characterized LRO biology, experiments in cultured fibroblasts provided much of the literature surrounding the biogenesis of enlarged lysosomes in *LYST* mutants. Other defining features characteristic of LYST-deficient fibroblasts include reduced exocytosis capacity and viability in response to membrane injury (64). Restoring *LYST* expression in beige fibroblasts reverses the enlarged lysosomal phenotype and rescues exocytosis, suggesting a possible link between lysosome size and cell function in fibroblasts (64). The baseline hyperactive inflammatory gene transcription was observed in CHS fibroblasts. However, the cells failed to initiate an appropriate immune response following lipopolysaccharide (LPS) challenge, indicating an inability to respond to an inflammatory stimulus. This defect was attributed to abnormal expression of toll-like-receptors 2 (TLR2) and TLR4 or aberrant intracellular processing or membrane localization of these receptors (88). This observation is similar to findings in innate and adaptive immune cell types, specifically NK cells, neutrophils, macrophages, and T cells. However, Westphal et al. noted that *LYST-*mutant dendritic cells and macrophages exhibit normal activation of MAPKs upon TLR4 stimulation, demonstrating that LYST likely does not interact with the MyD88 pathway (89). This observed dysfunction correlates with impaired TLR3- and TLR4-induced IRF3 phosphorylation, suggesting that TRIF pathway dysregulation is responsible for abnormal trafficking in *LYST-*mutant innate immune cells (89). This finding does not discredit Wang et al., but rather suggests that LYST likely exerts cell-type specific effects. Fibroblasts also provided the foundation for investigating LYST in conditions beyond CHS. Cutaneous wound healing studies showed that LYST is an important regulator of secretory exocytosis by fibroblasts, which confer deficits in MCP-1, IGF-1, and IGFBP-2 secretion when *LYST* is mutated (40). Such insights into fibroblast behavior underscore the complexity of LYST’s impact, paving the way for broader implications in various physiological processes and pathological conditions.

### 4.7 Monocytes and macrophages

Monocytes, macrophages, and their products comprise the population of cells responsible for debris clearance, tissue remodeling, and aspects of host defense. These cells exhibit diverse functions by undergoing differential polarization into various tissue-specific phenotypes, ranging from resident macrophages to recruited pro-inflammatory monocytic precursors (90). Differential activation of macrophages into classical M1 or alternative M2 types occurs via Th1/PAMP and Th2 signaling, respectively. These prototypical phagocytes, similar to neutrophils discussed above, exhibit marked functional deficiencies due to their reliance upon the endolysosomal network and LRO’s in the context of *LYST* mutations.

Recent insights reveal that macrophage phagocytosis relies on lysosomal degradation and recycling (91). Furthermore, there is a newfound understanding of the dependence of monocyte/macrophage signal transduction on intracellular membrane and endosomal trafficking events. Despite normal monocyte phagocytosis in both CHS patients and beige mice (92, 93), *Lyst*-mutant monocyte-derived granulocytes are still at an increased risk of bacterial infection (92).

Interestingly, studies indicate a delayed antitumor cytostatic and cytotoxic activity of beige macrophages against lung carcinoma during the initial 24-48 hours of exposure (93). These findings corroborate the notion that *Lyst* mutations interrupt, rather than prevent, trafficking of the phagolysosome. Recent findings suggest that Lyst mediates phagosome maturation and pro-inflammatory pathways in a Trif (TLR3 and TLR4)- dependent manner (89). Specifically, beige-J cells are not able to recruit Rab7 during late stage endolysosomal maturation, subsequently interrupting Trif-mediated pro-inflammatory signaling pathways (89).

### 4.8 Platelets

Platelets are important homeostatic and thrombotic regulatory cells. Following vascular insult, these cells often mark the first step of the wound healing cascade (94). Platelets contain LROs such as dense, alpha, and lysosomal granules. The LROs store effector molecules required for platelet aggregation, activation, paracrine signaling, and protein degradation (95). CHS patients classically present with platelet storage pool deficiencies resulting in bleeding tendency (96, 97). Though circulating platelet levels are normal, *LYST* mutations inhibit exocytosis of platelet dense granules. Bleeding tendency is a hallmark of the clinical presentation of CHS observed across species (17). Beige mice bleed excessively due to reduced ADP, ATP, and serotonin within platelet dense granules (98–100). Further study of beige platelets revealed an impaired ability to aggregate in response to collagen, thrombin, and phorbol-12-myristate 13-acetate stimulation, which the authors attributed to platelet storage deficiency (101). These results underscore the impact of dysregulated platelet granule exocytosis in the pathophysiology of CHS.

### 4.9 Melanocytes and pigment producing cells

Melanocytes are melanin-producing cells primarily located in the epidermis, cochlea, and iris (102). Melanosomes are specialized LROs that synthesize and contain the pigment melanin. Following maturation, melanosomes are anchored and transported to the cell periphery via microtubules, eventually transferring to adjacent keratinocytes from melanocyte dendrites (103). Studies reported enlarged and disorganized melanosomes in CHS melanocytes (104, 105). Tyrosinase catalyzes the production of melanin, and, along with beta-glucuronidase, is understood to be abnormally trafficked in cultured CHS melanocytes (105). Additionally, reports indicate reduced melanin quantity and uneven pigment distribution in the mouse retinal pigment epithelium, leading to eventual retinal detachment (106). This phenomenon is attributed to the accumulation of photoreceptor outer segment phagosomes, coinciding with elevated levels of cathepsins, MMP-3, and markers of oxidative stress (106).

Interestingly, pigment granule biogenesis in *Drosophila* is paired with machinery of lysosomal protein delivery (107). This evidence establishes a molecular explanation for commonly reported oculocutaneous albinism of CHS patients and the LYST-dependent melanosome/lysosome events required for normal skin, hair, and eye pigment distribution.

### 4.10 Mast cells

Mast cells actively mediate innate and adaptive immunity (108) by releasing histamine and other chemical mediators to modulate acute allergic inflammation (109). Dysfunction of mast cells is responsible for chronic allergic and inflammatory disorders, autoimmune diseases, and cancers (110). Large granules were observed in the peritoneal, dermal, and bone marrow derived mast cells of beige mice (11, 111, 112). Other studies demonstrated that, compared to WT rat mast cells, beige rat mast cells released relatively greater amounts of histamine, which appeared to cause granule enlargement (113, 114). In contrast to the secretory defect observed in other cell types, subsequent mast cell investigations demonstrated normal mast cell cytotoxicity in response to TNF-α stimulation in beige mast cells despite their enlarged granules (113). Mast cells have received relatively little attention in the LYST literature, perhaps due to their auxiliary role in the progression of CHS. However, mast cells represent an additional model system to study LYST function. The modulation of Lyst activity in mast cells could provide a promising alternative to current mast-cell targeted therapies. Specifically, mast cells have recently been implicated in the progression of inflammation-mediated neurodegeneration (115) and may play an underappreciated role in the progressive neurodegeneration observed in CHS survivors.

## 5 Subcellular morphology of LYST mutations: enlarged granules

Initial and continued pursuits toward understanding the effect of *LYST* mutations focused on the pathognomonic hallmark described at the initial discovery of CHS: stark changes in the morphology and function of secretory lysosomes.

Peripheral blood leukocytes from CHS patients underwent extensive examination using light microscopy and various staining techniques. Initially, the enlarged bodies in peripheral blood neutrophils were thought to be giant peroxidase granules, Döhle bodies, and azurophilic clumps (4, 5, 116). Subsequent technological advancements in electron microscopy and immunofluorescence led to the discovery that the enlarged bodies were, in fact, lysosomes, supported by the morphological characteristics, localization, and acid phosphatase activity (7–9). Further investigation extended this understanding to beige and CHS fibroblasts, revealing acid phosphatase-positive intracellular inclusions containing the lysosomal markers, alpha-2-macroglobulin and rhodamine (23, 24, 117, 118). Lysosomal origin was also confirmed in various cell types including beige osteoclasts, gastric chief cells, parietal cells, and renal proximal convoluted tubal cells with enlarged granules (119–121). Recent studies consistently affirmed the identity of the enlarged inclusions using specific lysosomal markers including lysosomal-associated membrane protein-1 (LAMP-1), LAMP-2, and Rab7 (40, 64, 87, 122–124).

Lysosomes are dynamic organelles that operate at the intersection of the endocytic and secretory pathways. Lysosomes originate from transport vesicles budded from the trans-Golgi network, and these vesicles periodically fuse with endosomes to degrade material. Early endosomes are specialized compartments that receive material from the extracellular environment through processes like endocytosis. Early endosomes transition into late endosomes, marked by a conversion of the small GTPase Rab5 to Rab7 (125, 126). Late endosomes fuse directly with lysosomes, forming a hybrid organelle that acts as a site for degradation. Lysosome populations reform from these hybrid organelles. Lysosomes undergo fusion with one another, other intracellular compartments, or target membranes facilitated by docking proteins, including SNAREs, Rab GTPases, and Rab effector proteins (127). Lysosomes are critical for macromolecular degradation and recycling, intracellular protein and vesicle trafficking, and cellular signaling (127). Lysosome related organelles (LROs) are similar to lysosomes, and some cells utilize LROs for lysosomal functions (128). While the LROs originate from the endolysosomal system, certain cells including neutrophils and melanocytes harbor specialized LROs such as azurophilic granules and melanosomes.

While the mechanisms of lysosome biogenesis are well-defined, the role of LYST in these processes remains incompletely understood. Lysosome numbers and size are maintained by a steady state of fission and fusion events (129). Dysregulation of fusion and fission manifests as morphological changes to lysosomes and LROs. Fusion with endosomes increases lysosome size by incorporating vesicle content and membrane material (28), while fission leads to fragmentation and size reduction. LYST is hypothesized to affect lysosomes and LROs by either i. limiting the fusion or ii. promoting the fission of endolysosomal organelles (23, 24, 26).

The first hypothesis is that the enlarged lysosomes arise from excessive fusion events (8, 22–26). Time course tracking of lysosomes in cultured CHS cytotoxic T lymphocytes demonstrated that over the course of 9 days, normal-sized lysosomes clustered around the nucleus and aggregated to form the characteristic enlarged structures found in *LYST* mutant cells (68). The greatest support for LYST’s potential inhibitory effect on lysosome fusion came from investigations into the *Dictyostelium* ortholog of LYST: large volume sphere B (*lvsB)*. In *lvsB*-null cells, the endolysosomal fusion and phagosome-phagosome fusion events were significantly increased as measured using a fusion assay (130). Further investigation found that *lvsB* antagonized the activity of the DdRab14 protein, a GTPase with pro-fusion activity (124). While the *lvsB-*mutant cells could not prevent the fusion of lysosomes with post-lysosomes, they did not affect the fusion of post-lysosomes with early endosomes (124). Post-lysosomes are secretory vesicles destined for exocytosis, indicating that the role of lsvB in inhibiting lysosome fusion is specific to certain cellular compartments and does not affect the fusion events involving early endosomes. This suggests a nuanced and targeted regulatory mechanism where lsvB selectively modulates lysosomal fusion processes, highlighting the complexity of intracellular trafficking and secretion pathways in the context of lvsB and DdRab14 protein activity. LYST-related fusion/fission dynamics were further explored by comparing *lsvB* null cells with two well characterized fission defect mutants, µ3-null and WASH-null cells. *lsvB-*null cells exhibited increased multi-particulate phagosomes compared to the fission defect mutants and WT cells. Tracking lysosome maturation with latex beads also showed a deceleration in particle transfer in the *IvsB* null cells. Ultimately, only lysosome fusion with post-lysosomes was impacted by absence of *lsvB* (124). In a follow-up examination, *IvsB* mutant revealed significantly enlarged lysosomes and decreased post-lysosomes. However, the authors were unable to claim whether fusion dysregulation caused the enlarged lysosomes (15, 131).

A competing model establishes LYST and its homologs as a positive regulator of fission events, the second hypothesis for LYST’s effects on lysosomes. Experiments in murine fibroblasts demonstrated that *Lyst* overexpression led to the reversal of the enlarged lysosomal phenotype, yielding smaller-than-normal lysosomes with no observed effects on lysosomal fusion (27). Since fusion was unimpacted, overexpressing murine *Lyst* may have corrected the diseased phenotype by promoting fission events. Beige bone marrow derived macrophages were treated with acetic acid to fragment lysosomes. Upon withdrawal, beige lysosomes re-fused with no delay compared to WT cells. Then, CHS and beige fibroblasts were treated with vacuolin-1 to cause lysosomal swelling. The swelled lysosomes in beige fibroblasts recovered slower compared to WT fibroblasts, yet overexpression of *Lyst* increased lysosomal size recovery (27). Findings from these experiments led the authors to conclude that mutations in *LYST* impact lysosomal fission, not fusion (28). Similar findings are also observed in a *C. Elegans* study, in which disrupting the *LYST* ortholog, *Lyst-1*, resulted in decreased lysosome size. To determine if this was attributable to defects in lysosome fusion, the authors created double knockouts for *Lyst-1* and *Cup-5*, which is a known lysosomal fission regulator necessary for limiting lysosome size. In *Cup-5* mutants, disrupting genes known to be involved in lysosomal fusion suppressed lysosome enlargement (73). The *Lyst-1/Cup-5* double mutant, however, did not experience a reduction in lysosome size, leading authors to conclude that Lyst-1 was not involved in lysosome fusion (14).

While other studies contributed additional insights into LYST-associated abnormal granules, they have not definitively concluded whether LYST mediates lysosome morphology through fusion or fission events. In the context of CHS, cytotoxic T lymphocytes exhibited abnormal localization of necessary effectors for lytic granule exocytosis, Muncl3-4, Rab27a, and Slp3, within enlarged intracellular organelles. Overexpression of these effectors restored degranulation in the CHS mutant cells, indicating that *LYST* mutations impaired the maturation of pre-secretory granules to secretory granules. However, the specific influence on lysosome fusion or fission events remained undetermined (123). In a *Drosophila* study investigating a LYST ortholog mutation, *Mauve*, researchers found that the enlarged lysosomal related organelle in *Mauve-*mutants resulted from uncontrolled late phagosome homotypic fusion prior to fusing with lysosomes. Ultimately, the data could not distinguish whether this was due to LYST regulation of fusion or fission events (13).

## 6 LYST function and cellular dynamics

The story of LYST and its place in cellular biology is enigmatic, characterized by extended gaps between publications. The compromised immune system that is characteristic of Chédiak-Higashi Syndrome is associated with enlarged lysosomes in various immune cells. While *LYST* mutations do not prevent activation of immune cells, the functional defects seem to be caused by impaired polarization of these enlarged lysosomes or granules to the immunological synapse (61, 132).

To explain the impaired polarization, some groups detailed apparent defects in microtubule (MT) organization in *LYST*-mutant cells. Oliver and colleagues investigated the formation of concanavalin caps on polymorphonuclear leukocytes (PMNs) and differences in capping behavior in WT and *LYST*-mutant mice (133). The study revealed that when various cell types are treated with concanavalin A (conA), lectin aggregates into one region of the cell, thereby forming a cap. Colchicine is an anti-mitotic agent that is known to disrupt microtubule polymerization and assembly, which is necessary for vesicle trafficking. PMNs from WT mice only cap with ConA after colchicine treatment, whereas PMNs from *Lyst-*mutant mice cap spontaneously. The similarity between *LYST-*mutant PMNs to colchicine-treated cells suggests a potential link between the mutant LYST phenotype and microtubule instability or dysfunction. Interestingly, this spontaneous surface cap formation was not unique to mice, as further studies indicated the abnormality in human peripheral blood PMNs from Chédiak-Higashi patients. This abnormality is linked to impaired microtubule function, which could be due to issues in 3’,5’ cyclic guanosine monophosphate (cGMP) generation. Indeed, treatment with cGMP decreased cap formation in *LYST-*mutant cells.

The investigation surrounding the role of LYST in microtubule function gained momentum with the discovery that beige mice exhibited an impaired mu-opioid and kappa-opioid receptor-mediated analgesic response to morphine (134, 135). This was one of the few studies that explored the role of LYST in neurological systems. The study discovered that impaired response to morphine was independent of receptor number or binding affinity, indicating the strain-dependency of the impaired response to morphine (136). The authors speculated that this was a consequence of disrupted membrane-related microtubular function in beige mice. Beige mice were given cholinergic agonists, which restored the analgesic response to morphine (137). This serves as the only evidence that the beige-specific impaired response to morphine was related to microtubular dysfunction. More recent studies provided definitive evidence that *Mauve* was necessary for microtubule nucleation during cell division (122).

While some studies support that the *LYST* mutations adversely affect microtubule function, other groups found intact MT function in addition to normal number, length, and distribution (138, 139). Despite normal MT function, both large WT lysosomes and abnormally enlarged lysosomes in *LYST*-mutant cells were unable to migrate through the cytoplasm due to their size (140). Thus, a new hypothesis emerged postulating that lysosome and granule dysfunction arose not from microtubule (MT) impairment but potentially from alterations in lysosomal motility due to abnormally enlarged size. This thought prompted further investigations into the intricate molecular mechanisms underlying *LYST* mutations and their broader implications in cellular processes.

Researchers further explored the role of LYST in lysosomal membrane dynamics. SNAREs (Soluble N-ethylmaleimide-Sensitive Factor Attachment Protein Receptors), are key proteins involved in membrane fusion processes, facilitating the docking and fusion of vesicles with target membranes. Some studies suggested that the connection between LYST and SNARE complex assembly and function could lead to abnormal lysosome morphology and lysosomal dysfunction observed in *LYST-*mutants. Yeast two-hybrid screening revealed several potential LYST binding partners including calmodulin (CaM), casein kinase II (CK2), 14-3-3, and Hepatocyte growth factor regulated tyrosine kinase substrate (HRS) (29). These proteins are well described members of the SNARE complex responsible for mediating cellular membrane fusion events such as exocytosis, vesicle transportation, and signal transduction (**Table 2**) (30). It is worth noting that additional experiments are necessary since the authors were unable to validate the binding partners in their study.

**Table 2:** Potential LYST binding partners.

Potential Binding Partner	Type	Function	Selected Cellular Processes
Casein Kinase II (CK2)	Constitutively active enzyme	Phosphorylates acidic proteins	Cell cycle control, DNA repair
Calmodulin (CaM)	Intermediate calcium-binding messenger protein	Senses calcium and regulates the activity of other proteins including protein kinases and ion channels	Cell signaling, lysosome fusion
Hepatocyte growth factor regulated tyrosine kinase substrate (HRS)	Endosomal protein	Required for trafficking receptor tyrosine kinases from the early endosome to the lysosome	Vesicular transport, lysosome biogenesis
14-3-3	Regulatory proteins expressed in all eukaryotic cells	Bind signaling proteins	Mitogenic signal transduction, apoptotic cell death, cell cycle control

The leading hypothesis was that LYST acts as a scaffolding protein facilitating critical interactions between SNARE complex proteins through regulation of Synaptotagmin and Protein Kinase C (PKC):

### 6.1 Regulation of synaptotagmin

Initially discovered in neurons, synaptotagmin is a ubiquitously expressed calcium sensing protein that mediates exocytosis. There are 17 different synaptotagmin isoforms in mice and humans (141, 142). Synaptotagmin VII mediates Ca^2+^-dependent lysosome exocytosis in fibroblasts, macrophages, and neurons (143–145). Phosphorylation of synaptotagmin VII by regulatory proteins modulates its Ca^2+^ sensing exocytic activity (146). Notably, CK2 has been shown to phosphorylate Synaptotagmin I at a site that is highly conserved across isoforms (147). Additionally, CaM seems to be required by synaptotagmin VII for vesicle trafficking to the plasma membrane (148). Beyond vesicle trafficking, synaptotagmin-mediated calcium-dependent exocytosis is also responsible for plasma membrane repair (149) (127). As LYST may act as a scaffolding protein for synaptotagmin-regulating proteins, these connections may explain the impaired plasma membrane repair and defective lysosomal polarization following plasma membrane injury observed in *LYST-*mutant cells (64).

### 6.2 Regulation of PKC activity

To further explain LYST involvement in vesicle docking and fusion machinery, it is necessary to consider other vesicle fusion proteins that interact with LYST’s potential binding partners, 14-3-3 and CK2. PKC is known to promote CK2 function, and PKC function is facilitated, in part, through interactions with 14-3-3 (150). Interestingly, beige cells naturally exhibited rapid downregulation of PKC activity. Beige NK cell cytotoxicity was improved upon treatment with an agent that prevents PKC breakdown (151). Additionally, this “PKC protecting” agent reversed the enlarged lysosome phenotype in beige cells; however, complete inhibition of PKC resulted in an enlarged lysosome phenotype similar to beige cells. PKC downregulation is potentially attributable to high levels of ceramide- a sphingolipid in beige fibroblasts which may cause the formation of enlarged granules (152). The relation of PKC downregulation to *LYST*-mutant phenotypes may loosely corroborate the relationship between LYST function and microtubules. Inhibiting PKC activity in WT cells enhanced ConA capping, similar to the abnormal spontaneous capping observed in beige cells (153). It is possible that PKC function is tied to cap formation, and that the downregulation of PKC activity leads to the abnormal capping in *LYST-*mutant cells. Despite the compelling case for PKC contributing to *LYST-*mutant phenotypes, some studies provide evidence against the link between PKC activity and enlarged lysosomes in *LYST-*mutant fibroblasts (154). Yet, it cannot be ruled out that while the coupling of PKC to enlarged lysosomes may not be universal, PKC isotypes may affect lysosome formation in other cell types, including macrophages and PMNs.

## 7 Toward a molecular understanding of LYST

LYST is a member of the class of “BEACH domain-containing proteins” (BDCPs) (31). The Beige and Chédiak-Higashi (BEACH) domain is a highly conserved region first identified within the LYST protein but present in all other BDCPs. BDCPs regulate a variety of cellular functions, such as synapse formation regulated by neurobeachin (NBEA) and granule size regulated by beige-like anchor protein (LBRA) and LYST (31). Other BDCPs are also involved in vesicle trafficking and membrane dynamics in humans (31). Mutations in BDCPs cause several complications including epilepsy (NBEA mutation), grey platelet syndrome (NBEAL2 mutation), and rare autoimmune diseases (LRBA mutation) (155–157). Current consensus suggests that BDCPs, including LYST, act as cytosolic scaffolding proteins that facilitate membrane events involved with vesicular trafficking (31).

Recent advancement in molecular technologies further promoted understanding of the molecular aspect of LYST. The LYST protein is comprised of 3801 amino acids (158), but alternative splicing yields multiple transcript variants (documented in RefSeq). Generally, LYST contains an ARM/HEAT domain followed by a ConA-like lectin domain, a pleckstrin homology-like (PH-like) domain, a highly conserved BEACH domain, and finally, C-terminal WD40 repeats (31, 159).

Even though current literature lacks studies into the biological functions of each domain, protein structural analysis revealed possible functions related to each domain. ARM/HEAT repeats have been shown to mediate vesicle trafficking and lipid binding (132, 160, 161). The PH domain has a central fold associated with membrane binding to specific proteins (162). The crystal structure of the 130-residue segment N-terminal to the BEACH domain reveals a backbone fold with a PH domain and an extensive interface between BEACH and PH domains (31). Consistent with these structural analyses, protein binding assays demonstrated that the PH and BEACH domains strongly interact with one another *in vitro* and *in silico* (32). LYST contains an 8-15 amino acid insertion in the BEACH domain that differentiates its function from the other BDCPs (31, 163). Interestingly, a Lyst-specific BEACH function that determines lysosome and granule size likely exists. In mouse cells, protein truncation of only the Lyst-BEACH C-terminal fragment, but not the Nbea-BEACH C-terminal fragment, led to reversible lysosome enlargement (31, 163).

**Figure 2:**
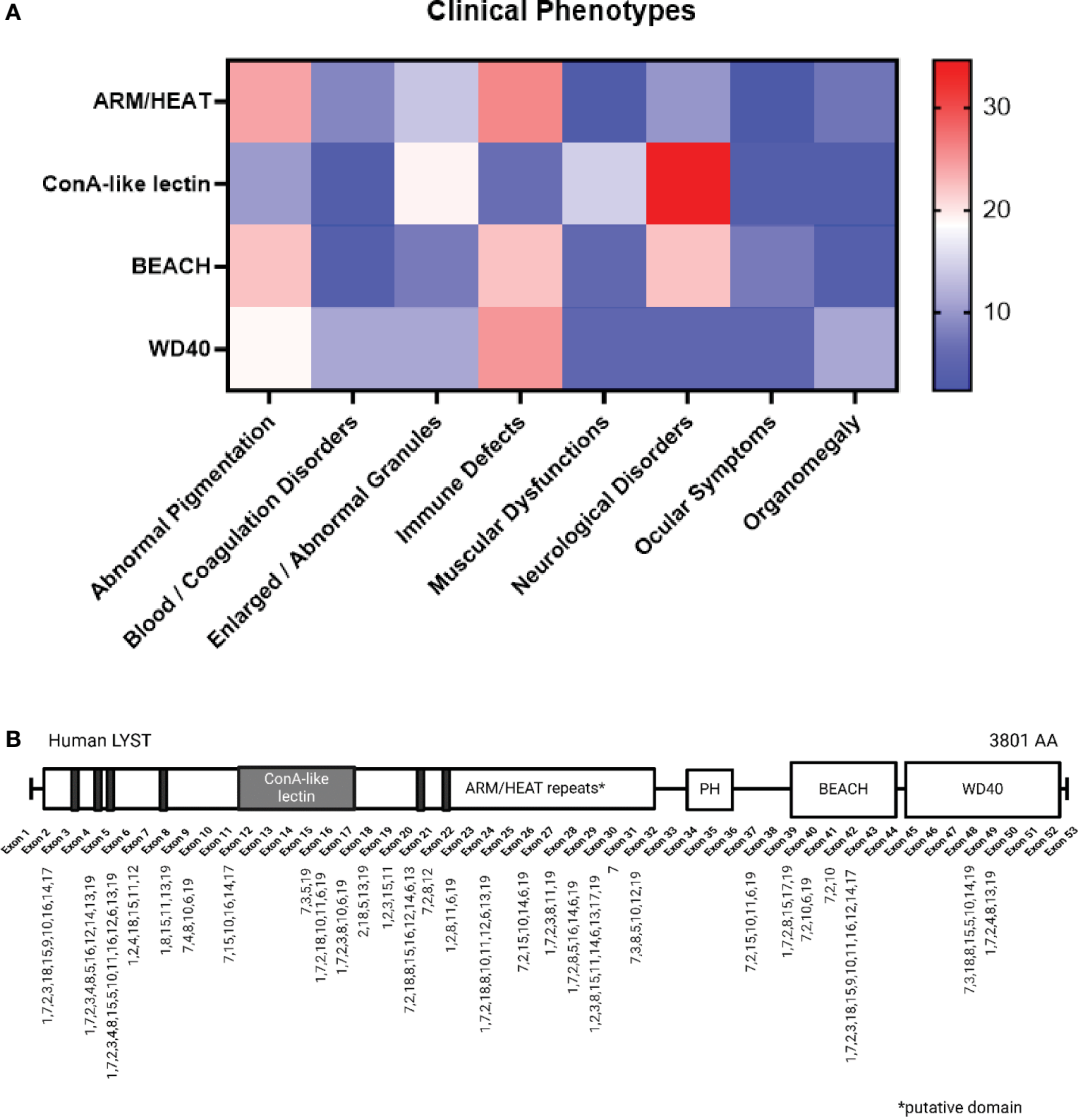
*LYST* Mutation and Correlation with Clinical Phenotypes. **(A)** The color gradient indicates the relative frequencies of specific disease phenotypes associated with each domain (e.g. Immune Defects account for 22.22% of cases with BEACH domain mutations). The 19 major phenotypes are categorized into eight groups and presented alphabetically, including: Abnormal Pigmentation (1. silver gray hair, 2. cutaneous albinism/partial albinism, 3. ocular albinism), Blood/Coagulation Disorders (4. coagulopathy/platelet dysfunctions, 5. isolated cytopenia, 6. pancytopenia), 7. Enlarged/Abnormal Granules, Immune Defects (8. fever/rash/lymphadenopathy, 9. oral infection/periodontitis, 10. defective/absent NK cell activity, 11. CMV/EBV seropositive, 12. respiratory infection, 13. recurrent infections), Muscular Dysfunction (14. motor dysfunction/myopathy/hypotonia), Neurological Disorders (15. GDD/intellectual disability/dementia, 16. neuropathy, 17. atrophic MRI brain), 18. Ocular Symptoms (i.e. myopia), and 19. Organomegaly (i.e. hepato/splenomegaly). **(B)** The mutation loci and their associated phenotypes are indicated on a schematic representation of the LYST protein spanning from exon 1 to exon 53, with a note indicating putative domains. Created with BioRender.com.

The parallel advancements in murine reagents and DNA sequencing contributed much of the genotype and phenotype correlations reported to date. A comprehensive literature review was conducted, involving a systematic search of peer-reviewed articles and case reports associated with “LYST” and “Chédiak-Higashi Syndrome” through the PubMed database (https://pubmed.ncbi.nlm.nih.gov/advanced/). We analyzed 29 case reports related to CHS, involving 61 patients (**Figure 2**, **Supplementary Table 1**) to explore the potential connection between *LYST* mutant genotypes and clinical manifestations of the disease. The genetic investigations suggest a link between the nature of the mutant *LYST* gene and the severity of the disease. For instance, it was observed that nonsense mutations, which typically result in the production of a truncated LYST protein, are associated with more severe phenotypes and often lead to infant mortality. In contrast, missense mutations tend to be linked to milder phenotypes or a later onset of the disease. A recent study by Morimoto and colleagues found comparable connections between the type of mutation and disease severity, providing additional support for genotype-phenotype correlations (164).

Further analysis indicates that the location of the mutated nucleotide(s) with respect to protein structure, in addition to mutation type, more closely correlates with disease phenotype (**Figure 2**). For example, mutations occurring within the ARM/HEAT repeats are associated with albinism, while neurological disorders are connected to mutations in the ConA-like lectin domain. Nonsense mutations within the WD40 domain are likely contributors to severe immune dysfunctions in CHS, leading to the accelerated phase, as depicted in **Figure 2**. Notably, ARM/HEAT mutations have been reported more frequently than mutations in other domains due to the domain’s larger size. However, a lack of data regarding the BEACH and WD40 domains may introduce potential inaccuracies in the interpretation of their functions.

Furthermore, it is important to highlight that the precise location of the ARM/HEAT repeats has not been definitively confirmed, despite proposed by Nagle et al. suggesting that these repeats may extend across a substantial helical region (31).

## 8 LYST and cancer

LYST was recently established as a gene of interest in patients with acute myeloid leukemia (AML), colorectal cancer, epithelial ovarian cancer, multiple myeloma, pulmonary carcinosarcoma, and sporadic chordoma (33–39). The association of *LYST* mutations with various forms of cancer is a chance discovery stemming from next generation sequencing analysis. Currently, the trend seems to be that downregulation or truncation of LYST protein leads to cancer progression. Downregulation of *LYST* was associated with low survival of AML patients, but in calcitonin receptor-like receptor (CALCRL) knockdown AML cell lines, *LYST* was upregulated (33). CALCRL deficient cells have been shown to be resistant to chemotherapy. The authors postulated that CALCRL downregulates *LYST* expression, which enlarges and stabilizes lysosomes and enhances drug resistance. In patients with sporadic chordoma, many patients carried truncating mutations in *LYST*. The authors explained that lysosomes were recently determined to be important for notochordal development, which could explain why defective LYST led to chordoma development. LYST also localized within copy number aberration regions in patients with multiple myeloma (39). Silencing *LYST* expression using anti-LYST siRNA inhibited proliferation and induced apoptosis in multiple myeloma cells (34). Both missense and nonsense *LYST* mutations were associated with pulmonary carcinosarcoma (38). Currently, studies are correlative but not mechanistic; authors appear to be divided on whether the connection of LYST to cancer is a spurious correlation or if *LYST* mutations truly drive cancer progression.

## 9 The future of LYST research

Chédiak-Higashi Syndrome (CHS) is a rare and life-threatening disorder, particularly affecting children. Despite identifying the causative gene decades ago, there is no comprehensive understanding of the cellular and molecular mechanisms underlying LYST function. The limited therapeutic options, primarily hematopoietic stem cell transplant, highlight the urgent need for more effective treatments to halt disease progression and prevent childhood mortality.

Recent studies employing various *LYST*-mutant organisms not only provided opportunities to study CHS, but also contributed to the understanding of the cell-specific role of LYST in endolysosomal trafficking and the immune response. Animal and clinical research highlights how *LYST* mutations manifest clinically in the setting of CHS, demonstrating its importance in immunity, neurological functions, pigmentation, and blood coagulation. Generally, cells from affected systems exhibit abnormal LRO morphology, impaired polarization, and decreased intracellular mobility, leading to reduced exocytosis of granules and protein trafficking.

While existing literature lacks unified theories on how LYST affects cellular processes, previous research proposes intriguing mechanisms contributing to the consistently observed enlarged lysosomes in CHS. This review provides a summary of the potential role of LYST in microtubule dynamics, granule fusion and fission events, and membrane docking facilitated by SNARE complex proteins. The current body of literature is divided on whether LYST impacts granule size through fusion or fission. Some studies suggest upregulated fusion events causing abnormal morphology, while others argue for insufficient fission as the culprit. The enlarged lysosomes and LROs do not fulfill their biological functions, and some studies suggest that the observed dysfunction involves changes in microtubule dynamics that impair proper vesicle transportation. Contradictory findings indicate intact microtubule function and suggest that the abnormally enlarged lysosomes physically cannot transit the actin cytoskeleton.

Sequencing technology enhanced our comprehension of the molecular structure and potential biological function of LYST by identifying genetic mutations. Recently, 11 novel disease-causing *LYST* mutations in the BEACH domain were identified using targeted Sanger sequencing, thereby expanding the scope and depth of LYST research (164). These findings provided valuable insight into mutation pathogenicity, laying the groundwork for potential CHS therapies and next-generation immuno-modulatory treatments.

Further investigation into the mRNA and protein sequences of LYST and lesser-explored members of BDCPs, such as WD repeat domain 81 (WDR81), is necessary to clarify the sequences and the precise locations of their domains. LYST and other BDCPs have diverse cellular functions, including cell cycle control, transcription regulation, apoptosis, and vesicle trafficking (31).

Knowing how LYST exerts its effects on a cellular and molecular scale holds significance not only for the development of CHS therapies but for other diseases involving cell regulation and vesicle trafficking. *LYST* mutations appear to be associated with certain cancer types, yet its potential role in cancer is not yet understood. Cutaneous wound healing studies revealed LYST’s crucial involvement in regulating secretory exocytosis by fibroblasts, which sheds light on the broader impact of LYST function (56, 57). Moreover, LYST attenuated immunomodulation combined with tissue engineering may improve transplant performance. While in the early stage of investigation, the importance of LYST in immune signaling suggests that LYST may influence the foreign body response to transplanted material. Thus, targeted alteration and temporary modulation of LYST function could offer immune privilege to recipients and protect donor tissues from overactive immune responses and rejection. The SCID/bg mouse model, where the Lyst beige mutation is introduced in SCID mice, has improved xenotransplantation compatibility (165). Recent advancements in tissue engineering, such as Tissue Engineered Vascular Grafts (TEVG) attracted attention due to the capacity for growth and remodeling (166). Despite their success, clinical studies have identified stenosis as a primary limitation. The immunodeficient SCID/bg mouse model demonstrated a drastic improvement in TEVGs patency (166). In the realm of regenerative medicine, LYST immunomodulation emerges as a promising target, aligning with the evolving paradigm of “immune interactive” strategies over traditional “immune evasive” approaches.

The discoveries presented herein underscore the potential for LYST as a tool for understanding the intersection of vesicle trafficking and cell cycle regulation and harnessing these findings to inform immune-modulatory strategies.

## Author contributions

MT: Conceptualization, Data curation, Formal analysis, Investigation, Writing – original draft, Writing – review & editing. JC: Conceptualization, Data curation, Formal analysis, Investigation, Writing – original draft, Writing – review & editing. GM: Investigation, Writing – original draft, Writing – review & editing. CK: Writing – review & editing. KB: Investigation, Writing – original draft, Writing – review & editing. SR: Writing – review & editing. JZ: Writing – original draft, Writing – review & editing. ChB: Conceptualization, Supervision, Writing – original draft, Writing – review & editing. CaB: Conceptualization, Data curation, Funding acquisition, Supervision, Writing – original draft, Writing – review & editing. JB: Conceptualization, Supervision, Writing – original draft, Writing – review & editing.
