# Supplementary material for: The lysosomal trafficking regulator “LYST”: an 80-year traffic jam
Source: Front Immunol. 2024 May 7;15:1404846. doi: 10.3389/fimmu.2024.1404846 (PMC11106369; doi:10.3389/fimmu.2024.1404846)
Supplement: Supplementary file 1 [file DataSheet_1.pdf]

Supplemental Materials

Table 1. *Clinical CHS phenotype / Lyst genotype associations*

Genotype ordered by affected codon(s) | Natural history of clinical manifestations

[Ref.]	Age (yr)	Sex (M/F)	Mutation	Zygosity	Type	Codon	Exon	Protein	Domain	Silvery grey hair	Enlarged / Abnormal Granules	Cutaneous albinism / partial albinism	Ocular albinism	Coagulopathy/Platelet Dysfunction	Ocular & Vision Symptoms (i.e. myopia)	Fever / Rash / Lymphadenopathy	GDD / Intellectual Disability / Dementia	Isolated Cytopenia (i.e. neutropenia)	Oral Infection / Periodontitis	Defective / Absent NK cell activity	CMV / EBV seropositive	Neuropathy	Respiratory Infection	Motor dysfunction / Myopathy / Hypotonia	Pancytopenia	Recurrent Infections	Atrophic MRI Brain	Organomegaly (i.e. Hepato / splenomegaly)
(1)	67	F	c. 2T>C ; c.433 C>T	Het	NR	1; 145	3; 5	p.Met1 ; p.Arg145	ARM/HEAT*	■	■	■					■		■	■							■	
(2)	1	F	NR	Het	Single-base Duplication	40	3	GCA → GGCA	ARM/HEAT*		■		■		■													
(3)	40	F	c.172C>G	Homo	Missense	58	3	p.Leu58Val	ARM/HEAT*								■					■		■			■	
(4)	<1	F	c.6676C>T; c.338_339delGTinsAGATCTTTGAGTGGA	Het	Frame Shift; Nonsense	2226; 113	5	p.R2226; p.S113Kfs	ARM/HEAT*	■	■	■																
(5)	21	F	c.961 T>C	Homo	Missense	258	5	p.C258R	ARM/HEAT	■	■	■											■					
(6)	5	F	c.925C> T	Het	Nonsense	309	5	p.R309X	ARM/HEAT*		■	■																
(6)	3	M	c.925C> T	Het	Nonsense	309	5	p.R309X	ARM/HEAT*		■	■														■		
(6)	16	M	c.925C> T	Het	Nonsense	309	5	p.R309X	ARM/HEAT*		■	■																
(7)	21	M	c.1507C>T; c. 9925G>A	NR	Nonsense; Missense	503; 3310	5; 43	p.R503X; p.G3309S	ARM/HEAT*		■			■								■		■		■		
(2)	<1	M	NR	Homo	Frame Shift	566	5	Single-base deletion	ARM/HEAT*				■					■										
(8)	NR	M	NR	Homo	Frame Shift	638	5	Premature p.638	ARM/HEAT*	■	■		■			■		■								■		■
(9)	NR	M	c.2413delG; c.10127A>G	NR	Frame Shift; Missense	805; 3376	6; 44	p.E805fsX806; p.N3376S	ARM/HEAT*; BEACH		■						■											
(10)	5.5	NR	c. 2620delT	Homo	Frame Shift	874	6	Truncation at p.874	ARM/HEAT*		■	■								■	■				■			■
(11)	5.5	M	c. 2620delT	Homo	Frame Shift	898	6	p.X898X	ARM/HEAT*		■			■		■		■										■
(2)	27	M	NR	Homo	Nonsense	1103	6	CGA→ TGA Transition	ARM/HEAT*			■					■					■				■		
(10)	2.5	NR	C3310T	Homo	Nonsense	1103	6	Truncation at p.1103	ARM/HEAT*		■	■					■			■	■				■			■
(12)	6	F	c.C3310T	Het	Frame Shift; Stop gain	1104	6	p.R1104X	ARM/HEAT	■	■		■				■						■			■		
(12)	1	M	c.C3310T	Het	Frame Shift; Stop gain	1104	6	p.R1104X	ARM/HEAT	■	■						■						■			■		
(13)	4.5	M	c.575insT	Homo	NR	NR	7	NR	ARM/HEAT*	■		■		■	■		■				■		■					
(14)	13	F	c.3726delG	NR	Frame Shift	1242	9	p.Lys1242 fs*25	ARM/HEAT*	■						■	■				■					■		■
(15)	6	F	c.3944-3945 ins C	Het	Frame Shift	1315; 1331	10	p.T1315fsX1331	ARM/HEAT*		■			■		■				■					■			■
(3)	63	M	c.4189T>G	Homo	Missense	1397	12	p.F1397V	(ConA)-like lectin		■						■					■		■			■	
(3)	53	M	c.4189T>G	Homo	Missense	1397	12	p.F1397V	(ConA)-like lectin		■						■			■				■			■	
(16)	58	M	c.4189T>G	Homo	Missense	1397	12	p.F1397V	(ConA)-like lectin		■						■			■				■			■	
(16)	53	M	c.4189T>G	Homo	Missense	1397	12	p.F1397V	(ConA)-like lectin		■						■					■		■				
(17)	4.5	F	c.5034_5038delTTGGT	Homo	Nonsense	1679	16	p.G1679Tfs*19	(ConA)-like lectin*		■		■					■										■
(13)	1	M	c.5004delA	Homo	NR	NR	17	NR	(ConA)-like lectin*	■		■			■													
(10)	1.3	NR	c. 5317delA	Het	Frame Shift	1773	17	Truncation at p.1773	ARM/HEAT*		■	■								■	■				■			■
(18)	4	F	c.5506C>T	Homo	NR	1836	18	p.Arg1836	ARM/HEAT*		■	■	■			■				■					■			■
(18)	<1	M	c.5506C>T	Homo	NR	1836	18	p.Arg1836	ARM/HEAT*	■	■		■			■				■								■
(15)	23	M	c.5541-5542 del AA	Het	Frame Shift	1847	18	p.Q1847fsX1850	ARM/HEAT*		■		■															
(15)	20	F	c.5541-5542 del AA	Het	Frame Shift	1847	18	p.Q1847fsX1850	ARM/HEAT*		■		■															
(15)	17	F	c.5541-5542 del AA	Het	Frame Shift	1847	18	p.Q1847fsX1850	ARM/HEAT*		■		■															
(19)	4	F	c.5719A>G; c.4863-4G>A	Het	Missense; intronic	1907	19	p.I1907V	ARM/HEAT*			■			■			■								■		■
(13)	3.5	M	c.5519 delC	Homo	NR	NR	20	NR	ARM/HEAT*	■		■	■				■				■							
(20)	2.5	M	NR	Het	NR	2026	21	NR	ARM/HEAT*		■				■	■	■						■		■	■		
(20)	63	M	NR	Het	NR	2026	21	NR	ARM/HEAT*		■	■										■	■	■				
(21)	2	M	c.6159_6160del	Homo	NR	2053	22	p.Met2053Ilefs*31	ARM/HEAT*		■	■				■							■					
(14)	5	F	c.6712C>T	Homo	Nonsense	2238	23	p.Arg2238	ARM/HEAT*	■		■				■					■				■			■
(22)	<1	F	c.7060-1 G>A	Homo	Stop codon	2355	25	L2355fsX2370	ARM/HEAT*	■		■			■	■							■		■	■		■
(10)	1	NR	c. del7060-7066	Homo	Frame Shift	2354	25	Truncation at p.2354	ARM/HEAT*		■	■								■	■				■			■
(10)	14	NR	c. 7555delT	Homo	Frame Shift	2519	27	Truncation at p.2519	ARM/HEAT*		■	■					■			■				■	■			■
(23)	<1	M	c.7645C>T	Homo	NR	2549	28	p.Q2549X	ARM/HEAT*	■	■	■	■			■					■							■
(24)	2	F	c.7786C>T; C.9106+1G>T	Het	Splice-site Mutation	2596	29; 38	p.R2596	ARM/HEAT*	■	■	■				■		■				■		■	■			■

(15)	21	F	c.7982C>G; c.8281A>T	Het	Nonsense	2661; 2761	30; 31	p.S2661X; p.R2761X	ARM/HEAT*				■				■							■		■		
(14)	<1	M	c.8080C>T	Homo	Nonsense	2693	30	p.Gln2693	ARM/HEAT*	■		■				■	■				■			■	■		■	■
(3)	33	M	c.8221C>T/ c.10374 +1G>T	Homo	Splice Site Mutation	2741	31	p.Arg2741	ARM/HEAT*		■																	
(25)	3	F	c.8380dupT	Homo	Frame Shift	2794	32	p.Tyr2794Leufs*8	ARM/HEAT*		■		■			■		■		■			■					■
(10)	<1	NR	Del9106-9161	Homo	Frame Shift	3036	38	Truncation at p.3036	PH_BEACH		■	■					■			■	■				■			■
(26)	<1	M	c.11183delA; c.9453delA	Het	Frame Shift	3151; 3728	40; 51	p. N3728fs; p. K3151fs	BEACH	■		■				■												■
(3)	49	M	c.4157delA /c.9476A>	Homo	Missense	3159	40	p.Asp1386Valfs*4/p.Asp3159Gly	BEACH								■										■	
(3)	46	F	c.4157delA /c.9476A>	Homo	Missense	3159	40	p.Asp1386Valfs*4/p.Asp3159Gly	BEACH		■						■										■	
(10)	<1	NR	c. 9590delA	Homo	Frame Shift	3197	41	Truncation at p.3197	BEACH		■	■								■					■			■
(10)	7.8	NR	9228 + 10bpins	NR	Frame Shift	3210	42	Truncation at p.3210	BEACH		■	■								■								
(13)	<1	M	c.9590delA	Homo	NR	NR	43	NR	BEACH	■							■				■							
(27)	43	F	c.9827_9832ATACAA; c.10701+8C>T	Homo	In-frame Deletion	3276	43	p.Asn3276_Thr3277del	BEACH			■	■		■		■		■					■				
(27)	38	M	c.9827_9832ATACAA; c.10701+8C>T	Homo	In-frame Deletion	3276	43	p.Asn3276_Thr3277del	BEACH						■		■		■			■		■			■	
(27)	33	M	c.9827_9832ATACAA; c.10701+8C>T	Homo	In-frame Deletion	3276	43	p.Asn3276_Thr3277del	BEACH						■									■				
(28)	<1	M	c.1540C>T ; c.9893delT	Het	Frameshift	3298; 3304	43	F3298fsX3304	BEACH	■	■		■		■					■			■					
(29)	<1	M	c.1102G>T	Homo	Nonsense	3668	50	p.E3668X	WD40		■		■		■	■	■	■		■				■				■
(5)	3	M	c.11362 G>A	Homo	Missense	3725	51	p.G3725R	WD40	■	■	■		■		■										■		■

1. Yarnell DS, Roney JC, Teixeira C, Freitas MI, Cipriano A, Leuschner P, et al. Diagnosis of Chediak Higashi disease in a 67-year old woman. Am J Med Genet A [Internet]. 2020 Dec 1 [cited 2023 Jan 10];182(12):3007–13. Available from: https://onlinelibrary.wiley.com/doi/full/10.1002/ajmg.a.61886
2. Nagle DL, Karim MA, Woolf EA, Holmgren L, Bork P, Misumi DJ, et al. Identification and mutation analysis of the complete gene for Chediak–Higashi syndrome. Nature Genetics 1996 14:3 [Internet]. 1996 [cited 2023 Jan 10];14(3):307–11. Available from: https://www.nature.com/articles/ng1196-307
3. Koh K, Tsuchiya M, Ishiura H, Shimazaki H, Nakamura T, Hara H, et al. Chédiak-Higashi syndrome presenting as a hereditary spastic paraplegia. J Hum Genet [Internet]. 2022 Feb 1 [cited 2023 Jan 10];67(2):119–21. Available from: https://pubmed.ncbi.nlm.nih.gov/34483340/
4. Fukuchi K, Tatsuno K, Sakaguchi K, Sano S, Sasaki T, Aoki S, et al. Novel gene mutations in Chédiak-Higashi syndrome with hyperpigmentation. J Dermatol [Internet]. 2019 Nov 1 [cited 2023 Jan 10];46(11):e416–8. Available from: https://pubmed.ncbi.nlm.nih.gov/31245861/
5. Sánchez-Guiu I, Antón AI, García-Barberá N, Navarro-Fernández J, Martínez C, Fuster JL, et al. Chediak–Higashi syndrome: description of two novel homozygous missense mutations causing divergent clinical phenotype. Eur J Haematol [Internet]. 2014 Jan 1 [cited 2023 Jan 10];92(1):49–58. Available from: https://onlinelibrary.wiley.com/doi/full/10.1111/ejh.12203
6. Al-Tamemi S, Al-Zadjali S, Al-Ghafri F, Dennison D. Chediak-Higashi syndrome: novel mutation of the CHS1/LYST gene in 3 Omani patients. J Pediatr Hematol Oncol [Internet]. 2014 [cited 2023 Jan 10];36(4). Available from: https://pubmed.ncbi.nlm.nih.gov/24072239/
7. Bhambhani V, Introne WJ, Lungu C, Cullinane A, Toro C. Chediak-Higashi syndrome presenting as young-onset levodopa-responsive parkinsonism. Mov Disord [Internet]. 2013 Feb [cited 2023 Jan 10];28(2):127–9. Available from: https://pubmed.ncbi.nlm.nih.gov/23436631/
8. Karim MA, Nagle DL, Kandil HH, Bürger J, Moore KJ, Spritz RA. Mutations in the Chediak-Higashi syndrome gene (CHS1) indicate requirement for the complete 3801 amino acid CHS protein. Hum Mol Genet [Internet]. 1997 Jul [cited 2023 Jan 10];6(7):1087–9. Available from: https://pubmed.ncbi.nlm.nih.gov/9215679/
9. Westbroek W, Adams D, Huizing M, Koshoffer A, Dorward H, Tinloy B, et al. Cellular Defects in Chediak–Higashi Syndrome Correlate with the Molecular Genotype and Clinical Phenotype. Journal of Investigative Dermatology [Internet]. 2007 Nov 1 [cited 2023 Jan 10];127(11):2674–7. Available from: http://www.jidonline.org/article/S0022202X15331924/fulltext
10. Certain S, Barrat F, Pastural E, le Deist F, Goyo-Rivas J, Jabado N, et al. Protein truncation test of LYST reveals heterogenous mutations in patients with Chediak-Higashi syndrome. Blood. 2000 Feb 1;95(3):979–83.
11. Dufourcq-Lagelouse R, Lambert N, Duval M, Viot G, Vilmer E, Fischer A, et al. Chediak-Higashi syndrome associated with maternal uniparental isodisomy of

chromosome 1. Eur J Hum Genet [Internet]. 1999 [cited 2023 Jan 11];7(6):633–7. Available from: https://pubmed.ncbi.nlm.nih.gov/10482950/
12. Jin Y, Zhang L, Wang S, Chen F, Gu Y, Hong E, et al. Whole Genome Sequencing Identifies Novel Compound Heterozygous Lysosomal Trafficking Regulator Gene Mutations Associated with Autosomal Recessive Chediak-Higashi Syndrome. Sci Rep [Internet]. 2017 Feb 1 [cited 2023 Jan 11];7. Available from: https://pubmed.ncbi.nlm.nih.gov/28145517/
13. Scherber E, Beutel K, Ganschow R, Schulz A, Janka G, Stadt U zur. Molecular analysis and clinical aspects of four patients with Chédiak-Higashi syndrome (CHS). Clin Genet [Internet]. 2009 Oct [cited 2023 Jan 11];76(4):409–12. Available from: https://pubmed.ncbi.nlm.nih.gov/19650863/
14. Gomaa NS, Lee JYW, el Sharkawy A, el Chazli YF, Hassab HMA, Doghaim NN, et al. Genetic analysis in Egyptian patients with Chediak-Higashi syndrome reveals new LYST mutations. Clin Exp Dermatol [Internet]. 2019 Oct 1 [cited 2023 Jan 11];44(7):814–7. Available from: https://pubmed.ncbi.nlm.nih.gov/30815890/
15. Tanabe F, Kasai H, Morimoto M, Oh S, Takada H, Hara T, et al. Novel Heterogenous CHS1 Mutations Identified in Five Japanese Patients with Chediak-Higashi Syndrome. Case Rep Med [Internet]. 2010 [cited 2023 Jan 11];2010. Available from: /pmc/articles/PMC3014749/
16. Shimazaki H, Honda J, Naoi T, Namekawa M, Nakano I, Yazaki M, et al. Autosomal-recessive complicated spastic paraplegia with a novel lysosomal trafficking regulator gene mutation. J Neurol Neurosurg Psychiatry. 2014;85(9):1024–8.
17. Singh A, Bryan MM, Roney JC, Cullinane AR, Gahl WA, Khurana N, et al. A clinical report of Chediak-Higashi syndrome in infancy with a novel genotype from the Indian subcontinent. Int J Dermatol [Internet]. 2016 Mar 1 [cited 2023 Jan 11];55(3):317–21. Available from: https://pubmed.ncbi.nlm.nih.gov/26499269/
18. Kaya Z, Ehl S, Albayrak M, Maul-Pavicic A, Schwarz K, Kocak U, et al. A novel single point mutation of the LYST gene in two siblings with different phenotypic features of Chediak Higashi syndrome. Pediatr Blood Cancer [Internet]. 2011 Jul 1 [cited 2023 Jan 12];56(7):1136–9. Available from: https://pubmed.ncbi.nlm.nih.gov/21488161/
19. Song Y, Dong Z, Luo S, Yang J, Lu Y, Gao B, et al. Identification of a compound heterozygote in LYST gene: a case report on Chediak-Higashi syndrome. BMC Med Genet [Internet]. 2020 Jan 6 [cited 2023 Jan 12];21(1). Available from: /pmc/articles/PMC6943916/
20. Möttönen M, Lanning M, Baumann P, Saarinen-Pihkala U. Chediak-Higashi syndrome: four cases from Northern Finland. Acta Paediatr [Internet]. 2003 Sep 1 [cited 2023 Jan 11];92(9):1047–51. Available from: https://onlinelibrary.wiley.com/doi/full/10.1111/j.1651-2227.2003.tb02575.x
21. Helmi MM, Saleh M, Yacop B, Elsawy D. Chédiak-Higashi syndrome with novel gene mutation. [cited 2023 Jan 11]; Available from: http://casereports.bmj.com/
22. Nielsen C, Agergaard CN, Jakobsen MA, MØller MB, Fisker N, Barington T. Infantile hemophagocytic lymphohistiocytosis in a case of chediak-higashi syndrome caused by a mutation in the LYST/CHS1 gene presenting with delayed umbilical cord detachment and diarrhea. J Pediatr Hematol Oncol [Internet]. 2015 Mar 6 [cited 2023 Jan 12];37(2):e73–9. Available from: https://pubmed.ncbi.nlm.nih.gov/25551669/

23. Wang M, Qu LJ, Wang J, Chen TP, Li Y, Xu Z, et al. [A novel mutation of the LYST gene in a Chinese family with Chediak-Higashi syndrome]. Zhonghua Er Ke Za Zhi [Internet]. 2019 [cited 2023 Jan 11];57(12):963–5. Available from: https://pubmed.ncbi.nlm.nih.gov/31795567/
24. Faber IV, Prota JRM, Martinez ARM, Nucci A, Lopes-Cendes I, Júnior MCF. Inflammatory demyelinating neuropathy heralding accelerated chediak-higashi syndrome. Muscle Nerve [Internet]. 2017 May 1 [cited 2023 Jan 11];55(5):756–60. Available from: https://onlinelibrary.wiley.com/doi/full/10.1002/mus.25414
25. Boluda-Navarro M, Ibáñez M, Liquori A, Franco-Jarava C, Martínez-Gallo M, Rodríguez-Vega H, et al. Case Report: Partial Uniparental Disomy Unmasks a Novel Recessive Mutation in the LYST Gene in a Patient With a Severe Phenotype of Chédiak-Higashi Syndrome. Front Immunol. 2021 Mar 31;12:1043.
26. Sun Y, Li Y, Hao J. Rare inclusion bodies within monocytes at accelerated phase of Chediak-Higashi syndrome. Clin Chem Lab Med [Internet]. 2018 Apr 25 [cited 2023 Jan 11];56(5):e105–7. Available from: https://pubmed.ncbi.nlm.nih.gov/29194041/
27. Weisfeld-Adams JD, Mehta L, Rucker JC, Dembitzer FR, Szporn A, Lublin FD, et al. Atypical Chédiak-Higashi syndrome with attenuated phenotype: three adult siblings homozygous for a novel LYST deletion and with neurodegenerative disease. Orphanet J Rare Dis [Internet]. 2013 [cited 2023 Jan 11];8(1). Available from: https://pubmed.ncbi.nlm.nih.gov/23521865/
28. Zarzour W, Kleta R, Frangoul H, Suwannarat P, Jeong A, Su YK, et al. Two novel CHS1 (LYST) mutations: clinical correlations in an infant with Chediak-Higashi syndrome. Mol Genet Metab [Internet]. 2005 Jun [cited 2023 Jan 11];85(2):125–32. Available from: https://pubmed.ncbi.nlm.nih.gov/15896657/
29. Manoli I, Golas G, Westbroek W, Vilboux T, Markello TC, Introne W, et al. Chediak-Higashi Syndrome with early Developmental Delay resulting from Paternal Heterodisomy of Chromosome 1. Am J Med Genet A [Internet]. 2010 Jun [cited 2023 Jan 11];152A(6):1474. Available from: /pmc/articles/PMC2947940/
